# 4079 Lessons learned from implementing Quality Improvement (QI) in academic clinical research setting

**DOI:** 10.1017/cts.2020.241

**Published:** 2020-07-29

**Authors:** Chin Chin Lee, DUSHYANTHA JAYAWEERA, Marjorie Godfrey, Matthias Salathe, Jonelle Wright, Ralph L. Sacco

**Affiliations:** 1 University of Miami Clinical and Translational Science Institute; 2 Dartmouth Institute; 3 University of Kansas Medical Center

## Abstract

OBJECTIVES/GOALS: We describe here the implementation of a pilot Quality Improvement (QI) program in clinical research processes in order to facilitate translation from bench to community. This presentation will also discuss challenges encountered by the research teams during the implementation of QI activities. METHODS/STUDY POPULATION: Miami CTSI collaborated with University of Kansas’ CTSA to test the implementation of a QI program for clinical research processes. The program has a duration of 1 year and consists of multi-modal training and coaching sessions with different research teams. Six teams comprising of Principal investigators, clinical coordinators, and regulatory specialists participated in the program based in applied clinical microsystem theory science. Team coaches and teams worked together to assess current processes, test new and improved processes, and standardize and disseminate applicable best practices of the QI program. RESULTS/ANTICIPATED RESULTS: The implementation of QI activities in large clinical research settings poses numerous challenges for the research team. We will present survey results from the coaching sessions and follow on feedback from the different teams involved in the program to implement the QI activities. We will describe the modifications and adjustments made to the original conceptual framework of QI program in order for it to be applicable and feasible for the settings of the University of Miami. We will provide recommendations for other academic clinical research centers that are considering implementing a QI program. DISCUSSION/SIGNIFICANCE OF IMPACT: The successful adaptation of a QI process to implement in academic clinical research settings relies on early engagement of the institution leadership, careful selection of team members, as well as developing communication skills to enhance team dynamics as a clinical research unit.

